# The digitally accountable public representation database: online communication by U.S. officials

**DOI:** 10.1038/s41597-025-05857-1

**Published:** 2025-09-25

**Authors:** Yuehong Cassandra Tai, Nitheesha Nakka, Khushi Navin Patni, Sarah Rajtmajer, Kevin Munger, Yu-Ru Lin, Bruce A. Desmarais

**Affiliations:** 1 Pennsylvania State University, PA University Park, 16802 USA; 2 European University Institute, Fiesole, FI 50014 Italy; 3 University of Pittsburgh, Pittsburgh, PA 15260 USA

**Keywords:** Politics, Government

## Abstract

We introduce the Digitally Accountable Public Representation (DAPR) Database, an innovative archive that systematically tracks and analyzes the online communication of federal, state, and local elected officials in the U.S. Focusing on X/Twitter and Facebook, the current database includes 28,834 public officials, their demographic information, and 5,769,904 X/Twitter posts along with 450,972 Facebook posts, dating from January 2020 to December 2024. The database integrates three interconnected datasets: metadata on elected officials, weekly aggregated X data, and weekly aggregated Facebook data. These weekly aggregated datasets provide detailed insights into platform activity, capturing officials’ posting volumes, engagement metrics, and content trends. Our framework ensures ongoing database expansion by incorporating new officials and platforms, maintaining its relevance and research utility for analyzing officials’ digital communication.

## Background & Summary

What elected officials say online is important. For better or worse, social media is central to 21st century democracy. Individuals who directly follow elected officials online form a small, relatively ideologically extreme, and highly politically active portion of the population^1^. Nevertheless, the online communications of public officials influence much larger segments of society through several indirect pathways. For example, officials’ online messaging shapes responses to public emergencies^2,3^, acts as an information source for the news media^4,5^, and facilitates interaction and deliberation with constituents and other stakeholders^6–9^. The importance of online engagement is even greater for subnational officials, who seldom attract national media attention and face limitations in statewide media coverage^10^. In understanding the effects of officials’ online rhetoric, consider, e.g., their online discussion of vaccination, which became a prominent and highly politicized topic during the COVID-19 pandemic. Public dialogue and attitudes toward vaccination are shaped by prominent figures’ online dialogue, including elected officials^11,12^.

We build the Digitally Accountable Public Representation (DAPR) database as a comprehensive resource for data and measurement related to U.S. elected officials’ online communication, as well as their gender, race, and demographic information. Our goal is to facilitate research and analysis by scholars but also journalists, activists, regulators, and voters. We are building the DAPR database towards coverage of all federal and state level elected officials in the U.S., as well as elected officials at the 100 largest U.S. municipalities. Following a recent line of work focused on state lawmakers’ online communications^5,8,13–17^, we piloted the DAPR database project using the online communications of state lawmakers. Despite their substantial size as a population of elected officials and their important roles in U.S. politics and policymaking, there is relatively limited scholarly attention to state lawmakers’ digital communication, especially in comparison to research on members of the U.S. Congress. The DAPR database helps fill this gap.

We have collected comprehensive data from 2020 onward, covering 28,834 public officials at the federal, state and local levels, their demographic information, and 5,769,904 X/Twitter posts along with 450,972 Facebook posts. We are releasing officials’ metadata along with weekly aggregated data on their platform usage, as well as aggregated follower and following data for select officials. The weekly-aggregated data includes the total number of posts per official by platform, weekly average engagement metrics, and term matrices capturing content trends in frequency of each word, #hashtag, and account mention within officials’ posts.

A number of studies have used DAPR data to examine state legislators’ responsiveness, online connections, misinformation dissemination, and discourse. Research analyzed X/Twitter posts from March 30 to October 25, 2020, and found that legislators’ attention to the COVID-19 pandemic correlates with state case numbers, national death counts, and local policies—with Republicans focusing on economic concerns and Democrats and Independents on public health^5^. Another study focusing on 300K interactions (following, retweeting, and mentioning behaviors) on X/Twitter revealed that legislators’ online networks are influenced by partisan, gender, racial, and geographic factors, with variations across and within states^18^. Other research using our X/Twitter and Facebook data shows that Republicans share 20 times more misinformation than Democrats on X/Twitter^19^ and that legislators in more professional states are less likely to share misinformation on Facebook^20^. A cross-platform analysis^21^ further indicated that sharing low-credibility content increases visibility on Facebook but decreases it on X/Twitter, with Republicans benefiting on both platforms and Democrats penalized on X/Twitter; also, uncivil language reduces X/Twitter visibility for both parties. Using DAPR data, these studies yield novel insights into digital politics and communication by revealing how state legislators’ online behaviors vary across partisan, demographic, and geographic lines, thereby deepening our understanding of elite political discourse in the digital age.

In the following sections, we provide an overview of our data collection procedures by using state legislators’ data as an illustrative case study. We then introduce three separate datasets that include information about officials at different levels. Officials’ metadata were subsequently validated by collecting human annotations on a small subset of the dataset to assess their reliability. We then outline procedures for data usage and integration with external datasets. Finally, we discuss our model for expanding and sustaining the DAPR database, including plans to incorporate new officials and platforms.

## Methods

### Data Collection

#### Official Data

**Fig. 1:**
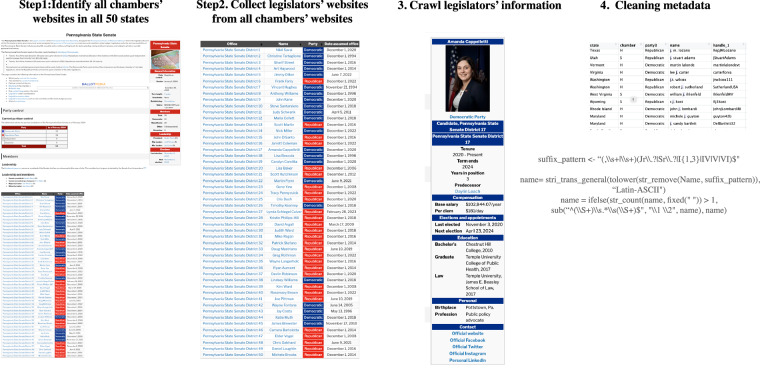
The Pipeline of Legislators’ Metadata Collection and Cleaning.

Taking state legislator data as an example, Fig. 1 illustrates our official data collection and processing workflow, broken down into four steps. In Step 1, we used Ballotpedia.org (https://ballotpedia.org/) as a starting point. Ballotpedia is commonly used as a data source in political science^22–24^. According to Ballotpedia^25^, it provides unbiased information on elections, politics, and policy, which is neutral and verifiable by its staff of writers, researchers, and election analysts. In addition, Ballotpedia employs a consistent framework for presenting information about elected officials across states over various periods. For example, legislators and their represented districts are listed on senate - and house-specific pages, from which individual legislators’ pages can be accessed. The combination of neutral information and consistent structure makes it a reliable and trackable source for our raw data collection.

In Step 2, from state chamber websites, we identified each legislator’s Ballotpedia webpage link. In Step 3, we collected available data on Ballotpedia, including each legislator’s name, chamber, office name, office level, office branch, district name, district type, partisanship, and their social media accounts. At the time of collection, legislators’ accounts on X/Twitter did not provide any identifiers indicating whether the account is private, official, or campaign, thus we collected all available account information associated with individual legislators. Legislators’ Facebook accounts provide account types, including private, official, and campaign. For office positions, we adopted Open Civic Data Division Identifiers (OCD-IDs) as district identifiers due to their ubiquity, predictability, and stability (https://open-civic-data.readthedocs.io/en/latest/index.html), which facilitates standardization across datasets.

To maximize the amount of available information, in Step 4, we carried out an extensive manual data entry. We consulted multiple sources for legislators’ demographic information, election information, and social media accounts from Google, Cook (2017)^14^, legislators’ official or campaign websites, and state chambers’ official websites.

Party affiliation and position may change over time due to shifts in partisanship or transitions between offices. For example, some individuals may move from serving as state legislators to holding local positions, such as school district board members, while others may get elected as representatives to a different chamber than where they initially served. To account for these changes, we manually verified their political party membership, the year of their election, and the start date of their term for state legislators who held office at any point during 2020 and 2022. We determined their party affiliation and office based on the affiliation and position legislators held for the longer duration during that period. When available from Ballotpedia, we included updated information reflecting changes in office and/or party affiliation. In addition to state legislators, most other officials who served at the federal or local level have their party and position information recorded through 2024. We will update our data regularly with timestamps to track different time periods in future iterations.

After Step 4, we conducted a thorough cleaning process using both automated and manual methods to remove noise from the scraped information and harmonize data that have been collected from different sources. Ballotpedia does not provide legislators’ gender and race information. Therefore, we employed both an automated and a hand-coding approach to identify gender and race. Regarding racial coding, we manually identified each legislator’s race based on seven racial categories outlined in the Candidate Characteristic Cooperative coding scheme^26^.

To identify legislators’ gender we applied the gender R package^27^, an algorithmic approach to inferring gender based on a large panel dataset of names collected from the U.S. Social Security database, spanning from 1932 to 2012. We also incorporated 811 hand-coded entries from Nakka (2025)^28^ and 1,714 entries from the Center for American Women and Politics (CAWP) Women Elected Officials Database^29^. However, a few gender and race variables may still be missing for officials at the federal and local levels, and we will update these as the project progresses. We also manually entered legislators’ districts, year of being elected, and vote share in the latest general election.

We generated a distinct identification number for each legislator. This unique ID links the metadata of public officials with content data from legislators’ X/Twitter and Facebook accounts. When combining our metadata with data from external sources, we encountered challenges due to discrepancies in the names of legislators and districts across different sources and over time. These inconsistencies, such as variations in the inclusion of middle names or prefixes, posed a significant obstacle to optimizing our dataset. As a solution, we implemented a comprehensive verification process that combined automated and manual coding methods. First, we programmatically merged datasets based on legislators’ names. This involved a fuzzy matching process where we manually adjusted names in other datasets to match those in our primary dataset, ensuring they represent the same individual. This automated matching process was repeated in multiple rounds for better accuracy. Next, we manually reviewed and verified the identity of any entries that could not be automatically merged using either full or fuzzy matching techniques in previous rounds.

#### Content Data from Social Media

Based on state legislators’ X/Twitter accounts, we collected legislators’ posts by using X/Twitter’s Official API v2.0 before rate limitations were imposed around June 2023. To maximize available data and reduce the effects of deleted posts afterwards, we collected the data, reaching back 7 days every day. When the data was stored, we cleaned the data by removing duplicates. In addition to basic information of posts, such as post ID, post time, and content, our raw data includes interactions such as the number of likes and retweets received by each post. Since May 2024, following the changes in June 2023, we resumed collecting posts under the new API terms. In this new round of data collection, we sampled posts of public officials at the state and local levels rather than conducting a full census. The latest X/Twitter data was recorded in December 2024. We also gathered state legislators’ follower networks approximately four times between 2020 and 2023.

For Facebook data, we collected state legislators’ data using CrowdTangle’s official API by following their terms of service. We collected publicly posted and available data in three rounds: April 2022, March 2023, and April 2023. However, many campaign or official accounts were no longer accessible during these three rounds of data collection. As with the X/Twitter data, metrics of interaction such as “likes”, “loves”, “haha”, and “angry”, as well as essential details regarding each post, have been collected.

In the documentation on Dataverse^30^, we present details on how changes in the APIs affect both our approach to collecting the data, and the overall coverage in the data.

Based on the collected raw data, we computed the aggregated weekly total number of posts and the average interaction per post for each official on each platform. We also created a TF-IDF(Term Frequency-Inverse Document Frequency)-weighted document-term matrix for weekly content using Python.

Since different preprocessing decisions could affect downstream inferences^31^, we intentionally omitted certain common steps—such as lowercasing, stemming, and lemmatization—when preparing our text data for the term matrices. The stylistic nature of social media content informed this decision. For example, all-caps words may convey emphasis or rhetorical intent. To preserve the original semantics and capture subtle cues often found in political elites’ posts, we implemented the following preprocessing steps. We first expanded contractions (e.g., “can’t”) into their expanded forms (“cannot”) to ensure consistency and reduce variability caused by different forms of the same word. We then translated emojis into corresponding descriptive words to capture emotional and contextual meanings.

To reduce noise, we replaced URLs with a placeholder and removed HTML tags. Although URLs may occasionally contain keywords or partial titles, they typically do not provide additional information beyond what is already present in the text. Instead, they often introduce noise into the feature space. Replacing them with a placeholder preserves their structural presence without emphasizing their content. HTML tags, as a markup language, do not contribute to the semantic meaning of the text and were therefore removed.

We split concatenated words written in CamelCase (e.g., “CamelCaseText”) into individual words (e.g., “Camel Case Text”) for stylized text. After that, commonly used words were removed using the standard English stopword list from the NLTK library. Before tokenization, we normalized whitespace, removed digits, and collapsed exaggerated character repetitions (e.g., “soooo coooool!”) to more standard forms (e.g., “soo cool!!”). We transformed the cleaned text into numerical feature vectors using the TF-IDF vectorizer. This approach helps capture the relevance of words while minimizing the impact of common, less informative terms. After generating the TF-IDF vectorized columns, we removed features (columns) that indicate corrupted or improperly decoded text, or consist entirely of non-letter characters (e.g., punctuation, numbers, or symbols). This step does not constitute full punctuation or number removal but filters out anomalous or uninformative features from the vectorized data.

Our preprocessing approach prioritizes retaining the original semantics and nuance of the text, while also ensuring reproducibility, allowing researchers and analysts to apply additional processing based on the specific goals of their studies.

Apart from the content of posts from the social media accounts of lawmakers, data on the relationships, followers, and accounts that legislators follow was also collected on X/Twitter. We aggregated the count of followers and followings for select legislators.

## Data Records

Current and future releases of DAPR dataset^30^ can be accessed via the DAPR Dataverse page (10.7910/DVN/A9EPYJ). Due to API restrictions, we cannot disseminate content data at the document level. Instead, we provide weekly metadata for content data. There are three types of files available on the DAPR Dataverse page:

• Metadata of Public Officials: Stored in a single CSV file, this contains detailed information about the public officials from different levels included in the dataset. In addition to officials’ demographics and political positions data, we also record the dates of each official’s first post on X and their first post on Facebook, and aggregate statistics on select officials’ followers and followings. The following and follower numbers are historical and static, collected before June 2023, and will not be updated. Table 1 shows the structure and variables of the officials’ metadata with variable types, column headers, data description, and source.

**Table 1:** Overview of Public Officials’ Metadata Information.

Type	Column Header	Description	Source
Unique ID	official_id	Unique identifier for each official	Authors’ coding
Demographics	name	Name of the official	Ballotpedia/Cook^14^/Wikipedia/Official websites
Demographics	firstname	First name of the official	Ballotpedia/Cook^14^/Wikipedia/Official websites
Demographics	lastname	Last name of the official	Ballotpedia/Cook^14^/Wikipedia/Official websites
Demographics	gender	Gender of the official	Nakka^32^ and Authors’ coding
Demographics	race	Racial or ethnic categorization of the official	Nakka^32^ and Authors’ coding
Demographics	party	Political affiliation	Ballotpedia/Wikipedia/Official websites
Official position	state	State abbreviation	Ballotpedia/Wikipedia/Official websites
Official position	state_fips	State Federal Information Processing Standards (FIPS) codes	United States Census Bureau
Official position	office_name	Office name	Ballotpedia/Wikipedia/Official websites
Official position	office_level	Office level (e.g., State, Federal, Local)	Ballotpedia/Wikipedia/Official websites
Official position	office_branch	Office branch (e.g., Legislative, Judicial, Executive)	Ballotpedia/Wikipedia/Official websites
Official position	district_name	District name	Ballotpedia/Wikipedia/Official websites
Official position	district_type	District type (e.g., State Legislative, Congress, State)	Ballotpedia/Wikipedia/Official websites
Official position	OCDID	Open Civic Data Division Identifiers	Open Civic Data Identifiers
Official position	yr_elected	Year in which the official was elected	Ballotpedia
Official position	vote_pct	Vote percentage in the latest general election	Ballotpedia
Official position	yr_vote	Year in which the latest election took place	Ballotpedia
Official position	bp_url	Ballotpedia URL	Ballotpedia
First post and Following data	first_post_fb	Date of first Facebook post in the database for the official	Authors’ calculation
First post and Following data	first_post_X	Date of first X/Twitter post in the database for the official	Authors’ calculation
First post and Following data	num_follower	Number of followers on X, available for select officials	Authors’ calculation
First post and Following data	num_following	Number following on X, available for select officials	Authors’ calculation

• Weekly Aggregated Data on X/Twitter by Public Officials: Thousands of CSV files store aggregated weekly data for each official, including their unique ID, total number of posts, list of shared post IDs, the average attention metrics, and term matrices. The average attention metrics include the average counts of likes, retweets, replies, and quotes received per post by week. TF-IDF-weighted document-term matrices track the relative importance and distribution of each word, #hashtag, and account mention by officials each week on X/Twitter. Table 2 illustrates variables of the weekly aggregated data with coressponding descriptions.

**Table 2:** Overview of Weekly Aggregated Data on X/Twitter by Public Officials.

Type	Column Header	Description	Source
Unique ID	official_id	Unique identifier for each official	Authors’ coding
Weekly Aggregated Data	calendar_week	ISO week and year (YYYY-WW)	Authors’ calculation
Weekly Aggregated Data	total_posts	The total number of posts made by the official in week XX of a given year YYYY	Authors’ calculation
Weekly Aggregated Data	urls	List of URLs to each post	Authors’ calculation
Weekly Aggregated Data	avg_likes	Average number of “Likes” received on the posts	Authors’ calculation
Weekly Aggregated Data	avg_retweets	Average number of “retweets” on the posts	Authors’ calculation
Weekly Aggregated Data	avg_replies	Average number of “replies” on the posts	Authors’ calculation
Weekly Aggregated Data	avg_quotes	Average number of “quotes” on the posts	Authors’ calculation
Term Matrices	#hashtag1	TF-IDF representation of each words, #hashtag, and account mentions based on content data by week	Authors’ calculation
Term Matrices	#hashtag2	TF-IDF representation of each words, #hashtag, and account mentions based on content data by week	Authors’ calculation
Term Matrices	…	TF-IDF representation of each words, #hashtag, and account mentions based on content data by week	Authors’ calculation
Term Matrices	term1	TF-IDF representation of each words, #hashtag, and account mentions based on content data by week	Authors’ calculation
Term Matrices	term2	TF-IDF representation of each words, #hashtag, and account mentions based on content data by week	Authors’ calculation
Term Matrices	…	TF-IDF representation of each words, #hashtag, and account mentions based on content data by week	Authors’ calculation

• Weekly Aggregated Data on Facebook by Public Officials: Thousands of CSV files store aggregated weekly data for each official, including their unique ID, total number of posts, list of post URLs, the average attention metrics, and term matrices. The average attention metrics include the weekly average counts of interactions such as likes, loves, angry, and care reactions, among others, received per post. These files also contain TF-IDF-weighted document-term matrices that capture the relative importance and weekly distribution of each word, #hashtag, and account mention by officials on Facebook. Table 3 displays an overview of variables of the weekly aggregated Facebook data.

**Table 3:** Overview of Weekly Aggregated Data on Facebook by Public Officials.

Type	Column Header	Description	Source
Unique ID	official_id	Unique identifier for each official	Authors’ coding
Weekly Aggregated Data	calendar_week	ISO week and year (YYYY-WW)	Authors’ calculation
Weekly Aggregated Data	total_posts	The total number of posts made by the official in week XX of a given year YYYY	Authors’ calculation
Weekly Aggregated Data	urls	List of URLs to each post	Authors’ calculation
Weekly Aggregated Data	avg_total_interactions	Average number of total interactions (likes, comments, shares, etc.) on the post	Authors’ calculation
Weekly Aggregated Data	avg_likes	Average number of “Likes” on the post	Authors’ calculation
Weekly Aggregated Data	avg_comments	Average number of “Comments” reaction on the posts	Authors’ calculation
Weekly Aggregated Data	avg_shares	Average number of “Shares” reaction on the posts	Authors’ calculation
Weekly Aggregated Data	avg_love	Average number of “Loves” reaction on the posts	Authors’ calculation
Weekly Aggregated Data	avg_wow	Average number of “Wows” reaction on the posts	Authors’ calculation
Weekly Aggregated Data	avg_haha	Average number of “Haha” reaction on the posts	Authors’ calculation
Weekly Aggregated Data	avg_sad	Average number of “Sad” reaction on the posts	Authors’ calculation
Weekly Aggregated Data	avg_angry	Average number of “Angry” reaction on the posts	Authors’ calculation
Weekly Aggregated Data	avg_care	Average number of “Care” reaction on the posts	Authors’ calculation
Term Matrices	#hashtag1	TF-IDF representation of each word, #hashtag, and account mention based on content data by week	Authors’ calculation
Term Matrices	#hashtag2	TF-IDF representation of each word, #hashtag, and account mention based on content data by week	Authors’ calculation
Term Matrices	…	TF-IDF representation of each word, #hashtag, and account mention based on content data by week	Authors’ calculation
Term Matrices	term1	TF-IDF representation of each word, #hashtag, and account mention based on content data by week	Authors’ calculation
Term Matrices	term2	TF-IDF representation of each word, #hashtag, and account mention based on content data by week	Authors’ calculation
Term Matrices	…	TF-IDF representation of each word, #hashtag, and account mention based on content data by week	Authors’ calculation

## Technical Validation

We performed a series of validation exercises for our officials’ metadata, updating and repeating these exercises in future iterations of the resource.

We first examined the data types in the columns of our data to ensure that the entered data conformed to the defined values. For example, the values in the yr_elected, vote_pct, and yr_vote column should be numeric. When setting the column types in R, if any missing values appeared, we double-checked to ensure that string variables from other columns were not mistakenly entered. We conducted the same test for columns containing string variables, such as OCD-ID, which represents districts that may be named purely by characters or by a combination of characters and numbers.

We also manually verified the value ranges. Regarding the time period range in the yr_elected and yr_vote columns, given that we collected state legislators’ metadata at the end of 2022, the values in these columns should not extend beyond that year. If any entries indicated a year of 2023 or later, we reviewed and corrected those errors. Similarly, we checked the range of vote_pct to ensure no values exceed 100. We thoroughly checked all columns containing time-related values and other specific ranges to confirm that all entries fell within the appropriate limits.

Regarding state legislators’ names, state, partisanship, district type (state legislative (upper) and state legislative (lower)), and district name, we sampled 500 entries and verified this information using officials’ Ballotpedia, Wikipedia, or official website pages. In this sample, we did not find any incorrect entries for names and states. Both party and district name variables demonstrated high intercoder reliability (*κ* = 0.99). The intercoder reliability of district_type was also high (agreement = 0.98), and the disparity stemmed from the chamber changes of nine legislators during the 2022 election. Since DAPR data is an ongoing project, these few mismatches will be solved automatically when we update the time period identifier for legislators.

The race and gender variables in state legislators’ metadata are validated by human coders. To validate race variables, we conducted a comprehensive process outlined in Nakka (2025)^28^ for each legislator, in which coders cross-referenced legislators’ self-identification on personal and professional websites, social media bios, Ballotpedia, as well as any racial or ethnic group memberships (e.g., the NAACP), photos, and name origins. Based on 500 samples, the intercoder reliability of race variable was high (agreement = 0.96).

For the gender variable, the gender R package^27^ was verified against hand-coding using a dataset containing legislators from ten randomly selected states, as described in Nakka (2024)^32^. According to that study, the gender R package performs well with high precision and recall scores ranging from 0.93 to 1. Nakka (2024) also notes, naming practices change over time, and many South/Eastern Asian, Middle Eastern, and North African names are underrepresented in the package’s database. Given that state legislatures have become increasingly diverse in the past decades, we hand-coded any missing values that the package could not identify. Specifically, missing values were checked independently and filled manually by human coders following Nakka (2025)’s gender identification cross-referencing procedure^28^. This process involved the verification of public officials’ pronoun usage and gender identification through legislators’ websites, Wikipedia pages, and/or Ballotpedia pages. During this process, the gender variable was routinely spot-checked to ensure accuracy. After coding all legislators, we sampled 200 entries and found no cases of mislabeling in gender.

## Usage Notes

We present a comprehensive venue for studying public officials and politics across two mainstream platforms since 2020. The goal of the DAPR project is to build publicly accessible data for a broad audience, including average citizens, researchers, media, and NGOs, to promote transparency, hold public officials accountable, and encourage civic engagement.

For users who are interested in understanding and analyzing the digital presence and behavior of U.S. elected officials, our datasets can be merged seamlessly using the unique official_id included in each dataset. When merging two weekly aggregated datasets, the time period identifier should also be used. The merged data allows both the public and researchers to monitor and evaluate politicians’ actions and responsiveness.

For users interested in merging our data with external datasets on politicians, individual-level data can be matched using politicians’ names, along with other demographic and political information from our metadata, and institutional-level data can be linked using party affiliation (party), state, district type, and/or OCD-IDs. Users should carefully select matching columns from our metadata, as different datasets often use inconsistent naming conventions for politicians.

For text-level data, we have accessed data, and continue to access data, via the official Facebook and X/Twitter APIs. The terms to which our access is subject to for both APIs prevent us from publicly disseminating all data in its completely raw form. To comply with these terms, we are releasing weekly aggregated data, including the number of posts, average engagement metrics, and term matrices. The weekly aggregated data includes dehydrated data containing lists of links to the posts shared by public officials during each week. Using these links, users can “rehydrate” this dataset to generate complete post objects containing the Facebook and X/Twitter text, engagement statistics, follower and following information, and more. Note that, in the event that a post has been deleted or is otherwise not publicly available, it will not be possible to access it using the URL.

While the DAPR project offers a valuable resource for studying public officials’ online behavior, it currently has several limitations. First, the emphasis on X/Twitter and Facebook excludes niche social media platforms with distinct user bases. Platforms like Parler or Truth Social might cater to specific political ideologies and potentially reveal different discourse topics, polarization levels, and online community interactions compared to mainstream platforms. Second, the current dataset has a temporal limitation regarding Facebook data. Facebook data only covers 2020 and 2021. This period coincides with significant events like the COVID-19 pandemic and the 2020 election, potentially influencing online political communication in a way not necessarily representative of broader trends. Given these limitations, users should interpret findings with caution, recognizing the dataset’s platform scope and temporal coverage when drawing conclusions or generalizing patterns.

We are addressing these limitations. The DAPR project is ongoing, with continuous updates and improvements. We are actively collecting posts from X/Twitter and Facebook and are expanding to include additional platforms such as YouTube, Instagram, and LinkedIn. Moreover, we are incorporating both national and local-level officials into the dataset. The compiled metrics of engagement and shared information from individual officials will be available for download in various formats on a daily and weekly basis to support research. This continuous endeavor to develop a time-series dataset across diverse platforms holds immense potential for scholars, fostering new perspectives on political research and the development of robust theoretical frameworks.

## Data Availability

The software tools used for data processing are described in the Methods and Technical Validation sections. The Python code used to generate the weekly aggregate data and term matrices, along with the R code for generating the gender variable, are openly available in our OSF repository^33^. Users can access the Python script titled scripts_data_aggregation and the R script titled script_gender_coding under OSF Storage in the Files tab.
